# Treatment effects of Shilajit on aspirin‐induced gastric lesions in rats

**DOI:** 10.14814/phy2.14822

**Published:** 2021-04-04

**Authors:** Naghmeh Ghasemkhani, Aidin Shojaee Tabrizi, Fatemeh Namazi, Saeed Nazifi

**Affiliations:** ^1^ Department of Clinical Studies School of Veterinary Medicine Shiraz University Shiraz Iran; ^2^ Department of Pathobiology School of Veterinary Medicine Shiraz University Shiraz Iran

**Keywords:** antioxidant, aspirin, gastric lesion, shilajit

## Abstract

The present study investigated the effects of Shilajit extract on aspirin‐induced gastric lesions in rats. We evaluated macroscopic and histopathological lesions in the stomach, measured the activity of oxidative stress enzymes in gastric tissue homogenates, and assessed serum electrolytes and parameters of kidney and liver function. Forty‐five male rats were allocated to five groups: Normal control, positive control, omeprazole treatment, Shilajit treatment, and Shilajit control. The treatment period lasted for four consecutive days. The size and number of gastric lesions were significantly reduced in the Shilajit and omeprazole groups compared to the positive control group, indicating a reduction in mucosal damage and the severity of edema and leukocyte infiltration in tissue sections. A significant increase was observed in the levels of all oxidative stress parameters, except malondialdehyde, in rats treated with Shilajit and omeprazole compared to those in the positive control group. The effect of the aqueous extract of Shilajit was comparable to that of omeprazole. These results indicated the protective effects of Shilajit against aspirin‐induced gastric lesions.

## Graphical abstract

This study demonstrated that Shilajit extract can alleviate aspirin‐induced gastric mucosal damage by increasing the activities of antioxidant enzymes. Therefore, it can be used as a gastric mucosal protective agent.

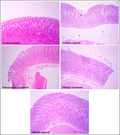

## 1 INTRODUCTION

Peptic ulcer is a gastric or duodenal mucosal lesion that develops at a point where the mucosal epithelium is exposed to aggressive agents. Although the etiology of peptic ulcer is still unclear, it is widely reported that peptic ulcers occur due to an imbalance between offensive factors and the maintenance of mucosal integrity through endogenous defensive mechanisms (Piper & Stiel, 1986). The pathogenesis of gastrointestinal disorders also includes oxidative stress and free radical damage. Non‐steroidal anti‐inflammatory drugs (NSAIDs) such as aspirin are recognized as one of the most common causative factors of gastric ulcers (Dehpour et al., 1994; Szabo, 1991). NSAIDs induce gastric ulcers by inhibiting prostaglandin synthesis and increasing the release of reactive oxygen species (ROS) and lipid peroxidation (Pohle et al., 2001; Shimada et al., 2004).

The available drugs for the treatment of peptic ulcer disease may result in adverse effects, drug interactions, and relapses (Srinivas et al., 2013). Hence, it is believed that herbal medicines are safer alternatives for the treatment of peptic ulcer disease due to their reported lower risk of relapse, side effects, and drug interactions compared to available chemical remedies (McQuaid, 2007). Plants are among the most desirable sources of new drugs and have been shown to have protective effects on gastric ulcers (Alkofahi and Atta, 1999).

Shilajit (also called Mumie) is a blackish to brown substance found in cracks and fractures of the mountains (Ghosal et al., 1991) and most of the hypotheses claim that it is formed by long‐term humification of the bryophyte plants (Ghosal et al., 1991; Schepetkin et al., 2003). Although Shilajit samples collected from different regions have been found to have varying percentage ratios of components, they have similar chemical composition and physical features (Garedew et al., 2004). Various studies have reported the effectiveness of Shilajit in the treatment of several diseases (e.g., gastrointestinal disorders), bone fractures, and wounds (Goel et al., 1990; Schepetkin et al., 2002). In addition, Shilajit has been reported to have several pharmacological properties, such as anti‐oxidative, anti‐inflammatory, anti‐ulcerogenic, and immunomodulatory effects (Agarwal et al., 2007).

The present study aimed to investigate the effects of Shilajit on Aspirin‐induced gastric lesions by evaluating macroscopic and histopathological lesions and assessing antioxidant and biochemical parameters.

## 2 MATERIALS AND METHODS

### 2.1 Plant material and extraction

Shilajit was purchased from Dr. Yousefi's Herbal Drug store and was identified and authenticated by a botanist from the Biology Department of Shahid Bahonar University of Kerman, Iran. Briefly, Shilajit was washed thoroughly with running tap water and cut into small pieces. To prepare the aqueous extract, 70 g of the sample was soaked with 1000 ml of distilled water on a shaker for 10 h at room temperature, filtered through a Whatman paper (No: 41), and finally freeze‐dried. The prepared extract was kept at −20°C until experimental use.

### 2.2 Study design

Forty‐five male Wistar rats, weighing 180–280 g, were used in this experiment. One week before the experiment, the animals were housed under optimal conditions of temperature (21–24°C) and light (12 h light/dark cycle) to adapt to the environment. During this time, they had access to water and standard rat pellets. Wister rats were randomly divided into five groups: NC (normal control, n = 5): animals received only normal saline for 4 consecutive days; PC (positive control, n = 10): animals received a single dose of aspirin (500 mg/kg, p.o.; Pars Darou Co.), followed by normal saline for 4 days (Sharma et al., 2011); OT (omeprazole treatment, n = 10): animals received omeprazole (20 mg/kg. p.o.; KRKA Pharmaceutical Company, Slovenia) for 4 days post‐administration of aspirin (Stephen et al., 2016); ST (Shilajit treatment, n = 10): animals received Shilajit (600 mg/kg. p.o.) for 4 days post‐administration of aspirin (El‐Sayed et al., 2012); SC (Shilajit control, n = 10): animals without ulcer induction received Shilajit (600 mg/kg. p.o.) for four consecutive days. Administration of omeprazole and Shilajit was done 1 hour after lesion induction in OT and ST groups, lasting for four consecutive days.

On the fourth day of the experiment, blood collection was performed under anesthesia with ketamine and xylazine. Then, after sacrificing all the rats in the study groups, their stomachs were immediately removed and opened along the greater curvature and their contents were drained into centrifuge tubes. Finally, the stomachs were gently washed with cold normal saline (0.9%).

### 2.3 The measurement of gastric pH

To determine whether the increased gastric pH had led to mucosal lesions, the pH value was measured 4 days after the administration of Shilajit and omeprazole. After laparotomy, the stomachs were removed and their contents were collected and centrifuged. The supernatants were used for pH measurements using an AD‐11 pH meter (Adwa Instruments).

### 2.4 Macroscopic evaluation

Macroscopic lesions were examined by a pathologist blinded to treatment allocation, and the surface of the glandular stomach, the sum of the area of the lesions, the percent of the lesion area, and the total number of lesions per glandular stomach were recorded and analyzed using Digimizer image analysis software (version 4.1; MedCalc Software) (Szabo et al., 1985). The lesions were classified into four groups as follows: 0 = normal mucosa; 1 = one to four small petechiae; 2 = five or more petechial spots or hemorrhagic streaks up to 4 mm; and 3 = erosions longer than 5 mm or confluent hemorrhages.

### 2.5 Histopathological evaluation

Tissue samples were fixed in 10% neutral buffered formalin, embedded in paraffin, sectioned at 5 μm, and stained with H&E. The histopathological evaluations were performed by pathologist blinded to treatment allocation.

### 2.6 Evaluation of oxidative stress parameters

The glandular part of the stomach was scraped and homogenized using 0.1 M phosphate buffer solution (pH = 7.4; 5 cc/g of tissue). Then, the suspension was centrifuged at 750 g for 20 min at 4°C, and the supernatant was used to determine the levels of malondialdehyde (MDA), and catalase (CAT), glutathione peroxidase (GPX), glutathione reductase (GR), and superoxide dismutase (SOD).

The level of MDA was measured based on its reaction with thiobarbituric acid in acidic conditions and high temperature (ZellBio GmbH). The color complex was measured calorimetrically at 535 nm and the results were expressed as mmol/mg tissue protein.

The CAT, GPX, GR, and SOD activities were measured using commercial kits (ZellBio Company) and expressed as the international unit per mg tissue protein.

### 2.7 Evaluation of biochemical parameters

Blood samples were centrifuged at 750 g for 15 minutes to isolate serum for the analysis of biochemical parameters. The following biochemical parameters in the serum samples were measured using commercial kits (Pars Azmun Co.) and a biochemical auto‐analyzer (Alpha Classic; Sanjesh Company): chlorine (Cl), phosphorus (P), sodium (Na), potassium (K), cholesterol, triglyceride (TG), glucose, blood urea nitrogen (BUN), alkaline phosphatase (ALP), aspartate transaminase (AST), and total protein (TP).

### 2.8 Statistical analysis

The data were expressed as mean ± standard error and analyzed by SPSS version 22 (one‐way ANOVA, post‐hoc Tukey's test, Kruskal–Wallis test, as well as Mann–Whitney test for comparison between two groups). *p* < 0.05 was considered significant.

## 3 RESULTS

### 3.1 pH measurement

**FIGURE 1:**
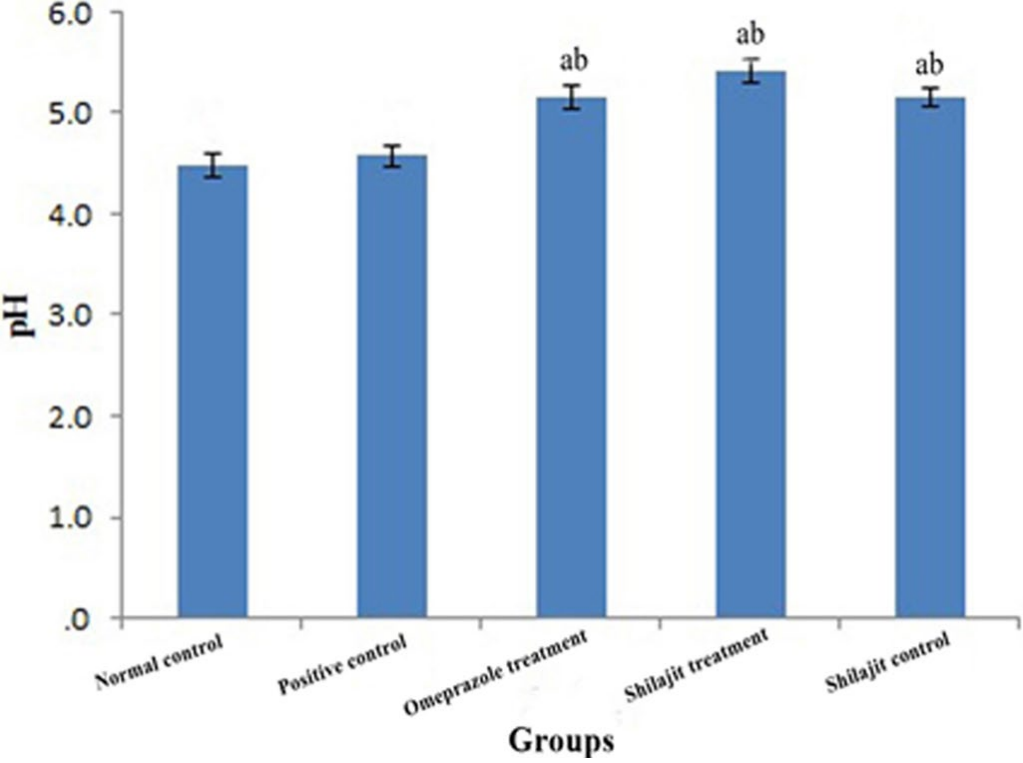
The pH of gastric contents in different groups (mean ± SEM). a: Significantly different from positive group at *p* < 0.05. b: Significantly different from normal group at *p* < 0.05

No significant difference was observed between the pH of the samples collected from the normal control and positive control groups. However, the administration of Shilajit extract and omeprazole caused a significant increase in the pH of gastric contents (*p* < 0.05) (Figure 1).

### 3.2 Macroscopic evaluation

**FIGURE 2:**
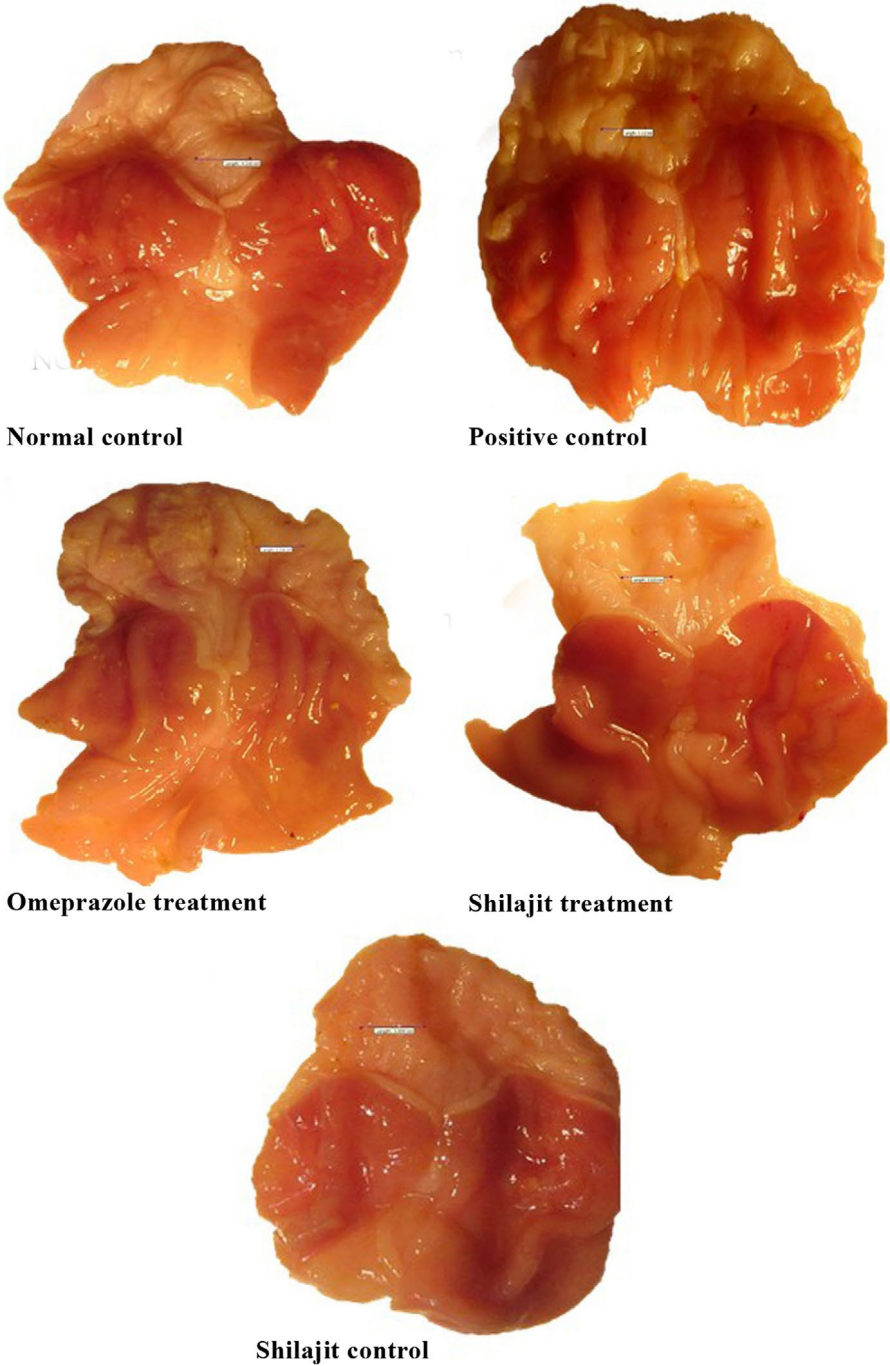
Macroscopic appearance of the stomach in different groups. Normal control group: scale bar = 4.21 mm; Positive control group: scale bar = 3.11 mm; Animals treated with omeprazole: scale bar = 3.31 mm; Animals treated with the aqueous extract of Shilajit: scale bar = 3.02 mm; Shilajit control group: scale bar = 3.60 mm

The macroscopic lesions observed in the study groups are shown in Figure 2. While no lesion was seen in the normal control and Shilajit control groups, the mucosal lesion area in the positive control group was measured to be 0.62 ± 0.21 mm^2^. The size and number of gastric lesions significantly decreased in both the omeprazole and Shilajit treatment groups in comparison with the positive control group (*p* < 0.05). The treatment effect of the aqueous extract of Shilajit was comparable to that of omeprazole since both decreased the gastric ulcer index. No significant differences were observed between the positive control and the treatment groups in terms of the lesion score (Table 1).

**TABLE 1:** Gastric lesion indexes among various groups (mean ± SEM)

Groups	N	Total surface area (mm^2^)	Total lesion area		Lesions		
			(mm^2^)	(%)	Score (0–3)		Number
					0–1 (%)	2–3 (%)	
Normal control	5	367.72 ± 20.33	0.00 ± 0.00^a^	0.00	0	0	0^a^
Positive control	10	373.19 ± 17.18	0.62 ± 0.21	0.16	10	90	193^b^
Omeprazole treatment	10	364.37 ± 13.25	0.17 ± 0.03^a^	0.04	30	70	81^a^
Shilajit treatment	10	327.42 ± 8.32	0.12 ± 0.02^a^	0.04	40	60	69^a^
Shilajit control	10	345.14 ± 9.91	0.00 ± 0.00^a^	0.00	0	0	0^a^

Note:

Data are presented as means ± SEM.

Abbreviation: N, number of rats/group.

^a^ Significantly different from positive group at *p* < 0.05.

^b^ Significantly different from normal group at *p* < 0.05.

### 3.3 Histopathological evaluation

**FIGURE 3:**
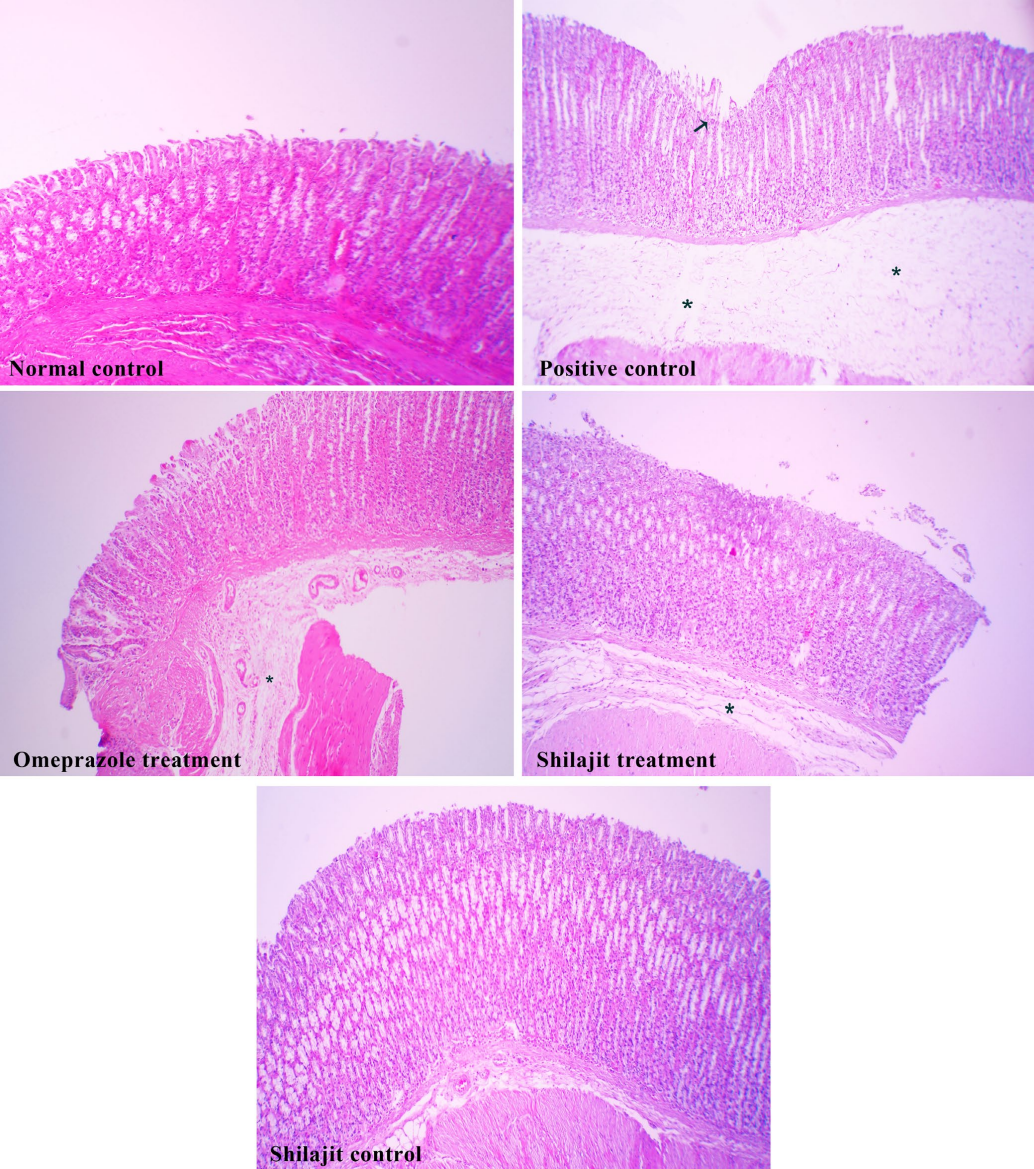
Histopathological evaluation of tissue sections. Normal control group: there was no lesion; Positive control group: moderate mucosal damage (arrow), with moderate submucosal edema (*) with mild leukocyte infiltration; Omeprazole and Shilajit treatment groups: a normal glandular pattern, with mild submucosal edema (*) and low leukocyte infiltration; Shilajit control group: there was no lesion. H&E, ×100

The gastric tissues of the normal control and Shilajit control groups were found to be intact with no lesions (Figure 3NC and 3SC) while the rats in the positive control group showed moderate mucosal damage, with moderate edema and mild leukocyte infiltration in the submucosal layer (Figure 3PC). In the Shilajit and omeprazole treatment groups, a normal glandular pattern was observed with mild submucosal edema and low leukocyte infiltration (Figure 3OT and 3ST).

### 3.4 Evaluation of oxidative stress parameters

The level of MDA and the enzymatic activities of CAT, GPX, GR, and SOD are shown as mean ± SEM in Table 2.

**TABLE 2:** Comparison of parameters value in the gastric tissue homogenate among different groups (mean ± SEM)

Groups	N	MDA (mmol/mg tissue protein)	CAT (IU/mg tissue protein)	GPX (IU/mg tissue protein)	GR (IU/mg tissue protein)	SOD (IU/mg tissue protein)
Normal control	5	0.56 ± 0.00^a^	0.69 ± 0.01^a^	379.18 ± 16.30^a^	45.26 ± 6.05^a^	42.03 ± 4.31^a^
Positive control	10	0.63 ± 0.01	0.50 ± 0.01	220.46 ± 9.21	17.58 ± 1.70	28.06 ± 1.06
Omeprazole treatment	10	0.59 ± 0.00^a^	0.61 ± 0.00^a^	367.75 ± 19.68^a^	38.14 ± 4.92^a^	44.03 ± 1.87^a^
Shilajit treatment	10	0.61 ± 0.00^b^	0.58 ± 0.01^b^	288.32 ± 28.27	28.14 ± 2.51	42.03 ± 1.57^a^
Shilajit control	10	0.57 ± 0.00^a^	0.64 ± 0.00^a^	403.48 ± 35.66^a^	30.53 ± 5.24	45.40 ± 2.20^a^

Note:

Data are presented as means ± SEM.

Abbreviations: CAT, catalase; GPX, glutathione peroxidase; GR, glutathione reductase; MDA, malondialdehyde; N, number of rats/group; SOD, superoxide dismutase.

^a^ Significantly different from positive group at *p* < 0.05.

^b^ Significantly different from normal group at *p* < 0.05.

The positive control group showed a significant increase in the level of gastric MDA compared to the normal control group. While MDA level decreased in the Shilajit treatment group, there was no significant difference between the Shilajit treatment group, on the one hand, and the positive control and omeprazole treatment groups, on the other hand, regarding MDA levels. The enzymatic activities of SOD and CAT significantly increased in the Shilajit and omeprazole treatment groups compared with the positive control group. However, no significant increase was observed in the activities of GR and GPX in the Shilajit treatment group compared with the omeprazole treatment and positive control groups.

### 3.5 Evaluation of biochemical parameters

**FIGURE 4:**
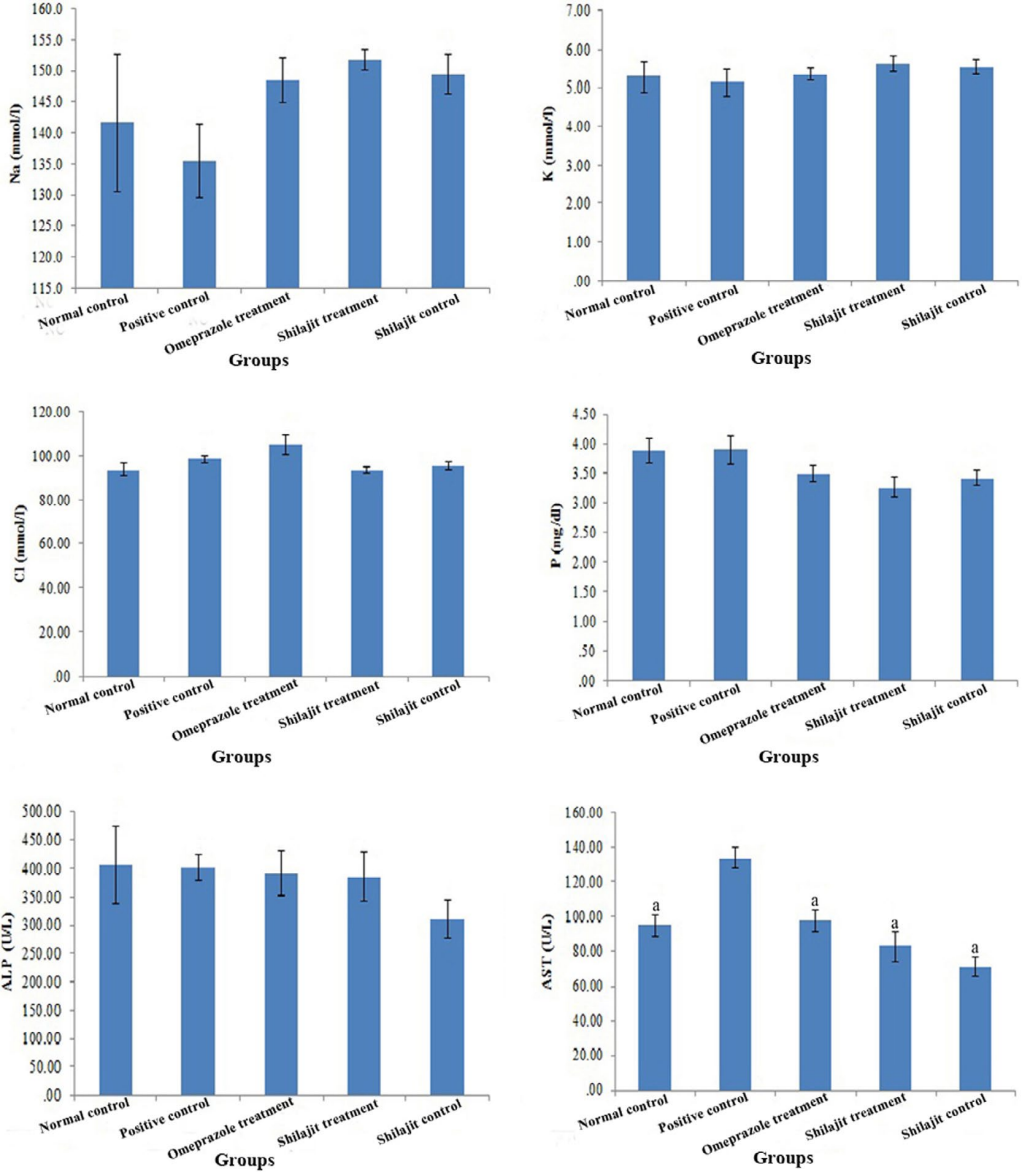
Na, K, Cl, P, ALP, and AST levels in different groups (normal control, positive control, omeprazole treatment, Shilajit treatment, Shilajit control), respectively (mean ± SEM). a: Significantly different from positive group at *p* < 0.05

**FIGURE 5:**
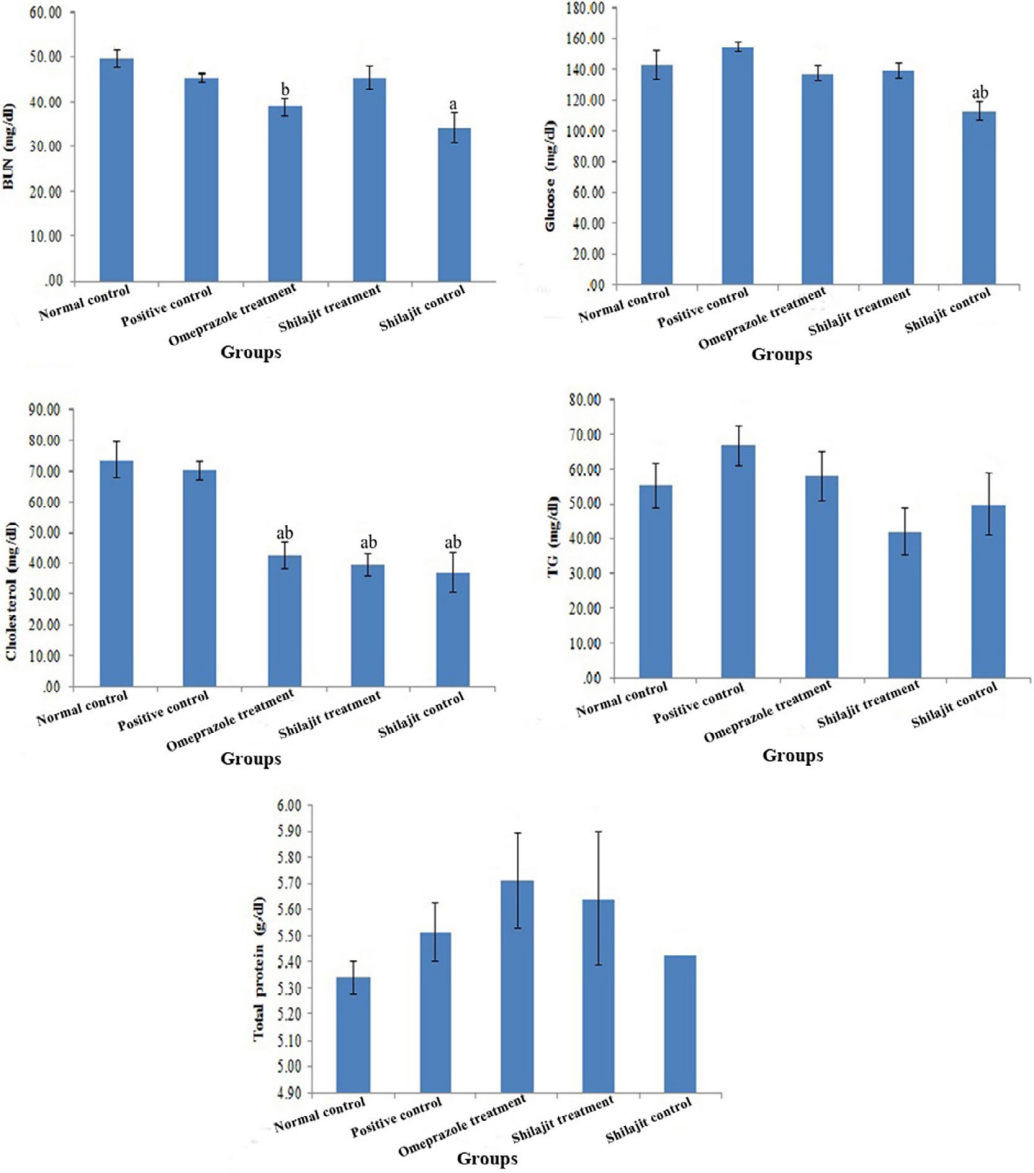
BUN, Glucose, Cholesterol, TG, and Total protein levels in different groups (normal control, positive control, omeprazole treatment, Shilajit treatment, Shilajit control), respectively (mean±SEM). a: Significantly different from positive group at *p* < 0.05. b: Significantly different from normal group at *p* < 0.05

The mean values (±SEM) of biochemical parameters are shown in Figures 4 and 5. The results showed that there were no significant differences between the study groups regarding the serum level of Na, K, Cl, and P (Figure 4a–d).

While there was no significant difference between the study groups regarding the level of ALP, the level of AST increased significantly in the positive control group compared to other groups. No significant difference was observed between the other study groups (Figure 4e,f).

BUN levels in the Shilajit control group decreased significantly compared to other groups, except for the omeprazole treatment group. Also, a significant decrease was observed in glucose level in the Shilajit control group compared to other groups (Figure 5a,b).

The level of cholesterol decreased significantly in the Shilajit control, Shilajit treatment, and omeprazole treatment groups compared with the negative and positive control groups. No statistical difference was observed between the positive and negative control groups in terms of cholesterol level. In addition, no significant difference was found between the study groups in terms of TG and TP levels (Figure 5c–e).

## 4 DISCUSSION

Gastric mucosal integrity is normally maintained by defense mechanisms. Several studies have reported a strong association between gastric mucosal damage and aspirin intake. Gastrointestinal ulceration and localized mucosal inflammation are among the most serious gastrointestinal complications (Goldstein & Cryer, 2015; Lucchi et al., 2012; Rafaniello et al., 2016). As an NSAID, aspirin is much more gastrotoxic than other NSAIDs and has been reported to cause mucosal damage, by inhibiting prostaglandin synthesis, and lipid peroxidation, by increasing the production of reactive oxygen species (ROS) (Beck et al., 1990; Pohle et al., 2001). In this regard, it is necessary to mention that oxidative stress is associated with increased production of ROS and reduced activity of antioxidant defenses (Schafer & Buettner, 2001). Thus, it seems that antioxidants can prevent the development of gastric mucosal lesions by scavenging ROS (Fahmy et al., 2015). In addition, aspirin administration has been reported to cause decreased gastric mucus secretion and increased gastric acid secretion (Hoogerwerf & Pasricha, 2001; Menguy & Masters, 1965; Singh & Debjani, 2012). Therefore, in developing pharmacological treatments to prevent or heal gastric ulceration, the side effects and the pathological aspects of these treatments should be taken into serious consideration (Lehmann et al., 2003). Hence, the aim of this study was to develop a new pharmacological treatment, that is, the aqueous extract of Shilajit, that could have gastro‐protective effects on aspirin‐induced gastric mucosal damage in rats.

In this study, both Shilajit extract and omeprazole were found to have anti‐ulcer activities, such as increasing the pH of gastric contents. The results of this study showed that treatment with the aqueous extract of Shilajit could have significant protective effects against aspirin‐induced gastric lesions. This finding is consistent with previous studies that have shown that Shilajit, as a rich source of phenolic compounds, can decrease acid and pepsin secretion, reduce the gastric ulcer index, stimulate cellular growth and repair, and increase mucin secretion and carbohydrate/protein ratio (Czinner et al., 2001; Goel et al., 1990; Rajic et al., 2001; Talbert, 2004). Omeprazole, as a proton pump inhibitor, has also been shown to protect gastric mucosa by inhibiting gastric acid secretion and thus increasing gastric pH (Shin & Sachs, 2008).

The size and number of gastric lesions significantly decreased in both omeprazole and Shilajit treatment groups compared to the positive control group. In addition, the rats in the positive control group showed moderate mucosal damage, with moderate edema and moderate leukocyte infiltration in the submucosal layer. Histopathological changes and gastric mucosal lesions in the stomach can be considered as key mechanisms in the pathogenesis of aspirin‐induced gastric mucosal injury. It is clear that inflammation and moderate leukocyte infiltration in the submucosal layer can also play an important role in the pathogenesis of gastric mucosal damage induced by aspirin/NSAID (Cheekavolu et al., 2016; Lee et al., 1992; Trevethick et al., 1993; Wallace et al., 1990). The disruption of the vascular endothelium can lead to increased vascular permeability, which, in turn, can cause edema and gastric mucosal lacerations (Patel et al., 2000).

In this study, the histopathological examination of gastric mucosa treated with Shilajit and omeprazole revealed a normal glandular pattern with mild submucosal edema and low leukocyte infiltration. The regeneration of gastric mucosa following the administration of Shilajit and omeprazole may be associated with the inhibition of oxidative stress.

In the present study, MDA, as the most widely used marker of oxidative stress, increased significantly in the positive control group compared to the normal control group. The Shilajit treatment group showed decreased MDA levels, accompanied by a decrease in the number of mucosal lesions; however, there was no significant difference between the Shilajit treatment group, on the one hand, and the omeprazole treatment and positive control groups, on the other hand, in terms of MDA levels. Shilajit has been found to have anti‐stress and anti‐anxiety effects (Frawley & Lad, 2004); thus, it can be used as a gastric mucosal protective agent. NSAIDs such as aspirin have been found to reduce the activities of CAT, SOD, and GPX (Das & Roy, 2012; Jainu & Devi, 2004). In this study, as expected, aspirin decreased the activities of antioxidant enzymes, but the administration of Shilajit and omeprazole increased their activities in the treatment groups compared to the positive control group. However, the increase was only significant for SOD and CAT activities. These antioxidant enzymes can reduce the production of ROS and have protective effects against lipid peroxidation (Radovanović et al., 2010). In addition, it is well known that Shilajit is a rich source of phenolic compounds, such as fulvic acids, benzoic acid 4′‐methoxy‐6‐carbomethoxy biphenyl (MCB), and tirucallane‐type triterpenoids, which give it antioxidant properties and enable it to act as a scavenger of ROS (Czinner et al., 2001; Goel et al., 1990; Rajic et al., 2001; Talbert, 2004). The results of this study revealed that the use of Shilajit, as an antioxidant, could increase the scavenging activities of antioxidant enzymes, especially those of SOD and CAT. As a result, it can be concluded that Shilajit has the potential to alleviate oxidative stress and aspirin‐induced gastric mucosal damage.

AST, a marker of liver injury, is a relatively nonspecific enzyme that is found in different tissues, including liver, kidney, heart, and skeletal muscle (Ozer et al., 2008; Voet & Voet, 2004). In this study, the increase in AST activity in the positive control group can be attributed to liver and kidney damage caused by high‐dose aspirin (Juhlin et al., 2008). Aspirin has been reported to cause a significant increase in BUN levels (Choi et al., 2010). The results of the present study showed that the administration of Shilajit resulted in a significant reduction in BUN levels in the Shilajit control group compared to other groups, except for the omeprazole treatment group. This effect of Shilajit extract could be due to two reasons. Firstly, as shown by Gupta (1966), Shilajit increases glomerular filtration due to its diuretic effect, leading to a decrease in urea level. Secondly, as some studies have reported, Shilajit has similar properties to anabolic steroids and thus its extract can reduce urea levels (Pandit et al., 2016). Another finding of this study was that the blood glucose level decreased in the Shilajit control group compared to other groups, indicating the hypoglycemic and anti‐diabetic properties of the Shilajit extract (Gupta, 1966; Talbert, 2004). Omeprazole has been suggested to be involved in cholesterol metabolism (D’Ugo et al., 2014). Similarly, Shilajit has been shown to reduce triglyceride and LDL cholesterol levels and increase HDL cholesterol levels (Sharma et al., 2011). These findings are consistent with the results of this study, in which treatment with the aqueous extract of Shilajit significantly reduced cholesterol levels in the Shilajit control, Shilajit treatment, and omeprazole treatment groups compared with the negative and positive control groups.

In conclusion, the study demonstrated that Shilajit extract ingestion is associated with reductions in aspirin‐induced gastric mucosal damage. This effect may be mediated by influences of Shilajit extract on the activity of antioxidant enzymes. Therefore, Shilajit has potential for further therapeutic development as a gastric mucosal protective agent in humans.

## CONFLICT OF INTEREST

The authors declare that there was no conflict of interest during this study.

## AUTHOR CONTRIBUTIONS

Authors’ contributions was Aidin Shojaee Tabrizi and Fatemeh Namazi in study design; Fatemeh Namazi, Saeed Nazifi, and Naghmeh Ghasemkhani in performing the study and analysis; and Fatemeh Namazi and Saeed Nazifi in preparing the manuscript. All authors read and approved the final manuscript.

## ETHICAL STATEMENT

The procedures were in accordance with the guidelines of Shiraz University for the care and use of laboratory animals.
